# Evaluation of Liver Function Tests and Risk Score Assessment to Screen Patients for Significant Liver Disease Prior to Bariatric and Metabolic Surgery

**DOI:** 10.1007/s11695-020-04486-4

**Published:** 2020-03-02

**Authors:** Anthony Antipass, Andrew Austin, Sherif Awad, David Hughes, Iskandar Idris

**Affiliations:** 1 School of Medicine, University of Nottingham, Nottingham, UK; 2 University Hospitals of Derby And Burton Foundation Trust, Derby, UK; 3 Division of Medical Sciences & Graduate Entry Medicine,School of Medicine, University of Nottingham, Royal Derby Hospital Centre, Uttoxeter Road, Derby, DE22 3DT UK

**Keywords:** Bariatric surgery, NAFLD,, Liver function

## Abstract

Bariatric and metabolic surgery is associated with significant improvement in obesity-related comorbidities, but for patients with non-alcoholic fatty liver disease (NAFLD), clinical outcomes are dependent on the severity of liver disease, i.e. improvement of NAFLD in most patients but increased risks of fulminant hepatic failure and/or bleeding varices in patients with more advanced cirrhosis. Our study showed that absolute values of liver enzymes were poor indicator of risk of liver fibrosis. The use of AST/ALT ratio, Fib 4 or NAFLD scores were appropriate screening tools, with each risk score appearing to pick out a certain phenotype of patients based on age, BMI or individual values of ALT, AST or platelet count. There is lack of agreement in some cases between FIB-4 scores and NAFLD scores when ruling out patients at high risk of liver fibrosis. Meticulous screening of patients at risk of liver fibrosis is crucial in order to reduce the risk of liver-related complications following bariatric and metabolic surgery.

## Introduction

Obesity is associated with a plethora of associated chronic comorbidities with non-alcoholic fatty liver disease (NAFLD) being the most common cause of chronic liver disease, with a prevalence of between 20 and 30% in the general population [1], increasing to up to 90% in severely obese patients [1]. NAFLD represents a spectrum of disease, of which a subset with more severe liver disease; namely, non-alcoholic steatohepatitis (NASH) may progress to cirrhosis [2, 3], where cumulative liver injury and necroinflammation result in fibrogenesis of the liver, associated with portal hypertension and hepatic synthetic dysfunction [3]. While bariatric surgery plays an important role in improving NAFLD, performing bariatric surgery in patients with undiagnosed NAFLD with advanced cirrhosis has been associated with severe repercussions, leading to sepsis, portal vein thrombosis, anastomotic leak, bleeding varices, fulminant hepatic failure and peri- and post-operative mortality [4–7]. Thus, careful pre- and post-operative screening, investigation, medical optimization and follow up of patients is crucial not only to identify individuals who would otherwise not be diagnosed to have advanced liver disease, but also crucially, to improve clinical outcomes, to determine the most appropriate surgical technique and modality and to prevent identification of advanced liver disease during surgery. While liver function test is commonly used as a screening tool to identify patients at risk of NAFLD, normal values even in the absence of stigmata of liver disease such as varices or ascites may not rule out advanced liver disease. Thus, Aspartate Transaminase/Alanine Transaminase (AST/ALT) ratio, in tandem with scoring systems such as Fibrosis (FIB)-4 and NAFLD scores are increasingly utilized to determine NAFLD patients at risk of liver fibrosis [8]. The FIB 4 score is a non-invasive liver fibrosis assessment based on patient age, platelet count, AST and ALT values, while NAFLD score includes additional variable such as body mass index, albumin and presence of glucose intolerance. Our investigation pathway thereafter dictates that patients with high scores will require transient elastography, FibroScan® assessment and patients with values that indicate high risk of liver fibrosis will require more definitive investigation such as liver biopsy and/or assessment of hepatic venous pressure gradient (8). Thereafter, all information will be available to the multi-disciplinary team to decide the overall risk and benefits of surgery.

## Methods

We undertook an evaluation study to determine the role, effectiveness and limitations of liver function assessment and risk scores to screen NAFLD patients at risk of liver fibrosis prior to bariatric and metabolic surgery. Retrospective data review of 392 patients who attended the bariatric medical clinic for consideration into bariatric surgery from January to July 2018 was performed. The study collected the data using the patient’s medical records including the demographics (age, gender, BMI and diabetic state) and blood test results (ALT, AST, albumin, platelets). Blood test results were only included if collected around the time of attendance (± 1 month). The data collected were then used to calculate the AST/ALT ratio, FIB-4 score and the NAFLD fibrosis score for each patient. Statistical analysis was carried out on all variables collected. All data were analysed using the independent T-test on SPSS software. The *p* value for significance was set at *p* < 0.05.

## Results

392 patients’ data who attended the clinics were analysed. The patient group included 114 males (29%) and 278 females (71%). The patient cohort was divided into a low-risk and high-risk group based on AST/ALT ratio of < 1 or > 1, respectively. Table 1 demonstrate the differences between the two groups. Interestingly mean AST and ALT levels for the low-risk group were significantly higher than those for the high-risk group. Furthermore, the group with elevated ratios also had a higher mean BMI than the overall group (49.3 versus 47.4 kg/m^2^, respectively) and were older (49.3 versus 46.3 years, respectively). By definition, the AST/ALT ratio, FIB-4 and NAFLD scores were significantly higher for the high-risk group compared to low-risk group.

**Table 1:** Comparison between the high risk and low risk groups based on AST/ALT ratio

Variables	Mean for 324 patients with ‘Low risk’	Mean for 68 patients with ‘high risk’	P value
AST (U/L)	27.2	24.4	*P* > 0.05
ALT (U/L)	37.9	19.3	P < 0.05
AST/ALT ratio	0.76	1.3	P < 0.05
FIB-4	0.75	1.09	P < 0.05
FIB-4 > 1.45	1.9	2.21	P < 0.05
NAFLD	− 0.47	0.44	P < 0.05
NAFLD > 0.676	1.54	1.87	P < 0.05
Age	46.3	49.3	P > 0.05
BMI	47.4	49.3	P > 0.05

### Agreement between FIB-4 Score and NAFLD Score

We looked at the number of patients with a normal FIB-4 score (< 1.45), but with an abnormal NAFLD score (> 0.676). It was found that out of 212 patients who had a normal FIB-4 score, 44 (20.6%) patients had an abnormal NAFLD score. Conversely, among patients with a normal NAFLD score (< 0.676), 2 (0.01%) out of 169 patients had an abnormal FIB-4 (> 1.45). Between these two discrepant groups, those with normal FIB4 but abnormal NAFLD score are significantly more obese, older with relatively normal AST and ALT. Therefore, the FIB-4 and NAFLD scores might not be able to be used interchangeably and that some patients with high risk of liver fibrosis on one score, maybe missed on another score. (Table 2) Agreement for normal values, however, appear higher when using NAFLD scores.

**Table 2:** Clinical and biochemical variables for different modalities to assess risk of liver fibrosis in patients undergoing bariatric surgery

Parameter	Mean for AST/ALT > 1	Mean for NAFLD > 0.676	Mean for abnormal NALFD but normal FIB-4	Mean for FIB-4 > 1.45	Mean for abnormal FIB4 but normal NAFLD
Number of patients	68	67	44	25	2
AST (U/L)	24.4	28.6	24.8	38.8	71.5
ALT (U/L)	19.3	31.7	30.3	39.2	94
AST/ALT ratio	1.3	0.99	0.93	1.08	0.73
Age (years)	49.3	54.7	51.2	60	42
BMI (kg/m^2^)	49.3	53.4	55.4	48.8	41.8
Albumin (g/dL)	37.2	36.4	36.1	37.0	38.5
Platelet (×10^9^/L)	277.7	238.2	263.1	188.4	162
FIB-4 score	1.09	1.31	0.9	2.06	1.69
NAFLD score	0.44	1.67	1.4	2.0	− 0.12

Comparison of individual demographic and biochemical parameters between high-risk categories based on AST/ALT ratio, FIB-4 score and NAFLD score is shown in Table 2. AST, ALT and age was significantly highest in patients who are considered to be high risk using the FIB-4 score, whereas BMI is highest in patients considered high risk using the NAFLD score. Platelet concentration was lowest in patients with high risk FIB-4 score. However, when investigating patients who are obese with relatively normal ALT and AST with abnormal FIB4 score, there an increased chance for these patients to have an abnormal value on the NAFLD score. Conversely, when assessing younger patients with lower BMI but significantly high ALT and AST values on NAFLD score, there is an increased chance of patients scoring abnormal on the FIB4 score.

## Conclusion

Assessment of advanced fibrosis during the course of NAFLD is vital in both practical and prognostic significance, particularly prior to bariatric surgery in order to avoid complications as a result of cirrhosis and particularly in patients with decompensated liver cirrhosis. In addition, recognition of the presence of liver cirrhosis will help surgeons to decide on which bariatric modality is most appropriate for patients – e.g. Roux en-Y gastric bypass which provides the most potential for weight loss, but may have a greater risk of vitamin deficiencies as well as rendering the stomach remnant and biliary tree inaccessible endoscopically in the event of a gastrointestinal bleed or biliary obstruction, versus sleeve gastrectomy which is technically less challenging and reduces the risk of malabsorption, but may predispose to bleeding risk in the setting of gastric varices [5]. Notable findings from the results of this review showed that an absolute value of liver enzymes per se does not predict risk of liver fibrosis, i.e. high-risk patients may have normal liver enzyme values. This is particularly so when using the FIB4 score in obese patients with normal ALT and AST levels to determine risk of liver fibrosis, where a high proportion of patients with normal FIB4 score in fact have abnormal risk score on the NAFLD score. Applying an AST/ALT ratio of > 1 facilitates categorising patients between high risk vs low risk, with previous studies reported an association between AST/ALT ratio > 1 and advanced fibrosis on liver biopsy [9, 10]. Comparing different modalities (AST/ALT ratio, NAFLD score and FIB-4 score) to determine individuals at high risk of liver fibrosis, we observed that abnormal FIB-4 scores is associated with the highest AST, ALT age values and the lowest platelet count, but abnormal NAFLD score is associated with the highest BMI. Lastly, using risk scores such as FIB-4 and NAFLD score have some limitations with regard to agreement for normal values (i.e., normal in one test, but abnormal in another test). Thus consideration needs to be made to perform both scores for patients with borderline values, to ensure patients at high risk of liver fibrosis is not missed on screening. Significant limitations to this study include that it took place in a single bariatric surgery centre creating selection bias, relatively small number of patients and the retrospective nature of the analyses. In addition, we did not have samples assessed for the Enhanced Liver fibrosis (ELF score) or the gold standard of liver biopsy to validate against our findings.

In conclusion, this study showed that liver enzymes alone without risk scores are an inadequate screening tool to identify patients at high risk of liver fibrosis. The use of AST/ALT ratio in combination with risk scores such as FIB-4 and NAFLD are appropriate screening tools to determine patients at high risk of liver fibrosis who requires further investigation. Each risk scores appear to pick out a certain phenotype of patients based on age, BMI or individual values of ALT, AST or platelet count. However, there is lack of agreement in some cases between FIB-4 score and NAFLD scores when ruling out patients at high risk of liver fibrosis. As such, we recommend that both scores need to be used where patients values are high or near threshold for abnormal values according to individual risk scores. Meticulous screening and post-operative follow up should take place, especially in patients with NAFLD so that they do not develop decompensation and suffer its complications due to undiagnosed cirrhosis as a result of bariatric and metabolic surgery.
