# Correction to: Evaluation of Liver Function Tests and Risk Score Assessment to Screen Patients for Significant Liver Disease Prior to Bariatric and Metabolic Surgery

**DOI:** 10.1007/s11695-020-04878-6

**Published:** 2020-07-30

**Authors:** Antony Antypas, Andrew Austin, Sherif Awad, David Hughes, Iskandar Idris

**Affiliations:** 1 School of Medicine, University of Nottingham, Nottingham, UK; 2 University Hospitals of Derby And Burton Foundation Trust, Derby, UK; 3 Division of Medical Sciences & Graduate Entry Medicine,School of Medicine, University of Nottingham, Royal Derby Hospital Centre, Uttoxeter Road, Derby, DE22 3DT UK

**Correction to: Obesity Surgery (2020) 30:2840–2843**

10.1007/s11695-020-04486-4

The name of author Antony Antypas was misspelled in the original article. It is correct here.

